# Studies on Sensory and Phytochemical Characteristics of Poppy (*Papaver somniferum* L.) Varieties for Their Oil Utilisation

**DOI:** 10.3390/foods12173165

**Published:** 2023-08-23

**Authors:** Katalin Gupcsó, Zoltán Kókai, Melinda Bálint, Szilvia Tavaszi-Sárosi, Éva Zámboriné Németh

**Affiliations:** 1 Department of Medicinal and Aromatic Plants, Institute of Horticultural Sciences, MATE, Villányi Str. 35-43, H-1118 Budapest, Hungary; kgupcso@gmail.com (K.G.); tavaszi-sarosi.szilvia@uni-mate.hu (S.T.-S.); 2 Sotiva Seed Ltd., Petőfi Str. 63/A, H-4440 Tiszavasvári, Hungary; 3 Department of Postharvest, Supply Chain, Commerce and Sensory Science, Institute of Food Science and Technology, MATE, Villányi Str. 35-43, H-1118 Budapest, Hungary; kokai.zoltan@uni-mate.hu (Z.K.); balint.melinda@uni-mate.hu (M.B.)

**Keywords:** *Papaver somniferum*, poppy oil, poppy flour, fatty acid, volatile compound, sensory profile, flavour, odour, SPME headspace

## Abstract

Poppy is a significant pharmaceutical crop, but the seeds and the cold-pressed oil have a big potential as healthy foods. Breeding has mainly focused on agronomical characteristics and alkaloid content. Here, we compared the sensory values, fatty acid and headspace volatile composition of poppy oils and flours produced from different varieties. Two industrial and four culinary varieties were cultivated in our field in 2021–2022. The sensory test differentiated well among varieties. Typical poppy odour and flavour were stronger both in the oils and the flours of the blue-seed varieties compared to the white-seed ones. For most varieties, the harvest year caused no relevant differences. Linoleic, oleic, and palmitic acids were the main components both in oils and flours. Larger differences were detected in the volatiles (e.g., 2 and 3 methyl-butanal and 3 methyl-butanol γ-n-caprolactone, pentofuran), depending on genotype and year. A higher ratio of saturated fatty acids negatively influenced the flavour and odour characteristics. In the headspace volatiles, these features correlated negatively with 2-pentylfuran and hexanal. The oil content of poppy flour has a positive effect on colour, appearance, tactility and poppy flavour. Our results support a goal-oriented use of poppy genotypes for high-quality dietary products.

## 1. Introduction

*Papaver somniferum* is one of our oldest and most important cultivated medicinal plants due to its many medicinal and industrial food uses. Poppy is cultivated in several countries for straw, which is an important pharmaceutical raw, and for seeds and fatty oils which are used in both alimentary and industrial production processes.

This multipurpose crop has a long history of domestication and breeding, resulting in the development of several different landraces, chemotypes and cultivars adopted to various uses and climatic conditions [1].

In recent decades, the selection work of *Papaver somniferum* focused on three main directions: creating cultivars accumulating rather high alkaloid amounts (above 2% in capsules dry weight) for industrial utilization, selecting for low alkaloid content for culinary usage (less than 0.1% morphine) and producing cultivars for special ornamental purposes with low alkaloid contents [2]. These purposes have been varied by the time and by the different countries.

The oil extracted from poppy seeds has already been mentioned both in the Bible and the Talmud, and it is considered equivalent to sunflower and olive oil, and it is therefore recommended for use on a larger scale [1,3,4]. The primary role of dietary fats in the human body is to provide energy; moreover, they are essential building blocks of living cell membranes, precursors of certain hormones, prostaglandins, and bile acids, and they provide the medium for the absorption, transport and storage of fat-soluble vitamins and other biomolecules. For humans, the consumption of certain vegetable or fish oils can provide essential fatty acids (linoleic acid, α-linolenic acid), which, beside their antioxidant effect, perform many special tasks in the body [5]. According to animal studies, poppy seed oil may improve antioxidant protection in the hippocampus after ischemia–reperfusion brain damage [6].

The cold-pressed poppy seed oil is light yellow in color, it has a pleasant taste and smell, and it is suitable for consumption. On the other side, the reddish-brown oil obtained by hot or post-pressing is used only for industrial and technical purposes [4,7] such as a solvent of oil paints while the iodized compound lipiodol may be used in radiological diagnostics [8]. It is doubtless that the biological and nutritional value of the poppy seed is considered favorable, which is related to its chemical composition [1].

Poppy seeds contain large amounts of minerals important for the human body—calcium, magnesium, potassium, iron and also carbohydrates, vitamins (B and E), trace elements (selenium, copper), as well as fiber, flavor, and fragrance substances [9].

Poppy seeds are small and vary in color from light yellow to deep blue/black. According to the studies revealing the possible correlation between the seed color and the fatty acid content, the blue-seed-type varieties usually contain less fatty acid content than the white or other color types [10]. However, as investigations of this issue include a small spectrum of genotypes, well-established conclusions could not have been taken before.

Although a relatively large number of studies have been published about poppy oil, only a few of them have been focusing on the volatile compounds, the sensory properties of the oil and only one [11] has analyzed poppy cake as well. According to the data, volatiles were considerably influenced by pressing methods but remained widely unaffected by storage time. As an example, a long-time-stored gray poppy seed oil sample from 1868 still showed all the characteristic of gray poppy seed oil [12]. In a Turkish study, 75 different volatiles were qualified. The authors concluded that the pre-treatments applied before cold pressing had a significant effect on the sensory properties of the poppy seed oils. Among the treatments, seed roasting was identified as the best processing operation in terms of improved sensory quality of the poppy seed oils [13]. From three Slovakian (one white- and two blue-seed) poppy varieties, 23 odour-active compounds have been found, of which hexanal, hexanol, 2-pentylfuram, hexanoic acid and pentanol represented the main compounds [14]. In a recent study, from the 44 detected volatile compounds, the most abundant ones were caproic acid (1.4–148 μg g^−1^), hexanal (0.9–15.2 μg g^−1^), 1-hexanol (0.3–20.1 μg g^−1^), limonene (1.3–9.4 μg g^−1^), and 2-pentylfuran (1.0–7.8 μg g^−1^) [15].

To modify the properties of products for the best consumer satisfaction, sensory analyses can be combined with aromatic analysis and/or consumer tests. Quantitative descriptive analysis and flavour profiling are the most widely used tests for the sensory characterization of oils and to assess consumer needs [16,17]. To achieve a better understanding of the sensory quality of poppy seed oil samples, Krist and co-workers [12] evaluated them by a sensory panel and by SPME-GC-MS (solid-phase microextraction–gas chromatography–mass spectrometry). The samples included grey, white and blue poppy seeds. Oils were pressed at room temperature (20 °C), except in the case of blue seeds where they were pressed at 20 °C in one case and 60 °C in another treatment. The evaluation of the odor and flavor character was made by a sensory panel, which consisted of 10 experts. They have collected those attributes, which were the most typical to the odor and flavor of the different oil types. Grey poppy seed oil was characterized by fatty, nutty and sweet odor, while in flavor, it showed nutty, sweet and peanut aftertaste notes. White poppy seed oil had a walnut, hazelnut, peanut and green odor accompanied by walnut and green flavor. Blue poppy seed oil pressed at 20 °C was nutty, had a pea peel odor, and a fatty, peanut and full-bodied flavor. When the pressing was made at 60 °C, fatty, green and roasted odors were identified. The flavor of the sample was fatty, roasted, nutty and green. In this study, the attributes were collected, but their intensities were not measured and compared by sensory profile analysis. Thirteen poppy seeds and their oils were analyzed in another study, five of them were winter varieties, and eight ones summer varieties [15]. Altogether, 22 attributes were used for the description of seeds and oils. Four of these attributes were only applied to seed samples (homogeneity of size, coarse, crunchy, and firm), three to oils (transparency, viscous, and oily) and the rest of the descriptors for both categories (poppy-typical, nutty, fatty/oily, rancid, green, hay like, mushroom like, musty, earthy, sweet, flowery, bitter, astringent, color, and homogeneity of appearance). Intensities were measured on a 5-point scale, where 0 was undetectable and 5 intense. For some visual attributes, RAL color reference cards were provided. Sessions were performed in sensory booths, two tablespoons of seeds or 15 mL of oil was served in glasses. The green aroma was associated with three methoxypyrazines that were exclusively found in summer poppies, and the sensory panel classified samples in particular by their fatty/oily, rancid, sweet, and green characteristics.

Nowadays, health promotion and disease prevention play a key role in human nutrition; therefore, the demand for poppy varieties with a favorable oil content and fatty acid composition also seems to be growing. In the experiment presented here, we studied the organoleptic properties of poppy oils and flours to detect consumers’ preferences for these valuable nutraceuticals. We compared samples originating from different poppy varieties and vegetation years to recognize the influence of these factors on the sensory and biochemical characteristics of the products. We also evaluated the headspace volatiles and fatty oil composition of the samples to reveal the genotype-specific characteristics as well as detect the eventual correlations of sensory features with fatty oil and volatile composition.

## 2. Materials and Methods

### 2.1. Plant Material, Growth Conditions and Sampling

In this study, we examined six different varieties of poppy. The plant material contains five registered varieties (‘Albakomp’, ‘Morgana’ Korona’, ‘Orel’, Zeno Plus’) and a candidate variety (‘Snow White’) one. Among them, two were industrial cultivars and four others selected for culinary purposes. The industrial variety ‘Morgana’ is characterised by morphine as the main alkaloid component while the other one ‘Korona’ has been selected for high noscapine content; both produce blue seeds. Three of the culinary genotypes (‘Orel’, ‘Albakomp’ and ‘Snow White’) are white seeded and one (‘Zeno Plus’) is blue seeded

**Figure 1:**
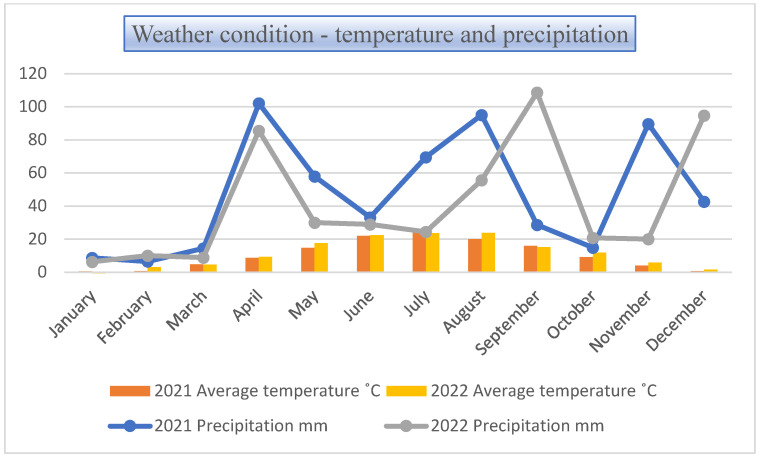
Monthly precipitation (mm) and average monthly temperatures (°C) in the experimental years 2021 and 2022.

The poppy varieties were grown in a small plot (17 m^2^) design in four replicates, both in 2021 and 2022 in Tiszavasvári, Hungary (GPS coordinates: latitude 47.9661886° longitude 21.4082049°). ‘Orel’ and ‘Albakomp’ were cultivated only in 2021. The soil type of the field is chernozem formed on loess which has a good humus content (2.71%) and a slightly acidic, near-neutral pH (6.42). The total monthly precipitation and average monthly temperature in 2021–2022 are shown in Figure 1. As a result of the unfavourable cold and uneven precipitation distribution in spring, the ‘Orel’ and ‘Albakomp’ varieties were destroyed in 2022.

Sowing of the overwintering poppy genotypes (‘Snow White’ and ‘Zeno Plus’) was performed by a small plot seeder in the middle of September and for the other varieties in the middle of March. Plant care was carried out according to the usual agrotechnology of poppy in Hungary. Pre-emergence weed control was provided by systemic herbicide Callisto 480 SC (mesotrione) 0.3 L/ha and Laudis OD (tembotrione) 2.25 L/ha was used post-emergently. Against insects, Karate Zeon 5 CS (lambda-cyhalothrin) was applied. Capsules were harvested at the stage of full ripening by hand in mid-July. The seeds were cleaned and stored in paper bags at room temperature until the analyses. Samplings for laboratory and sensory evaluations were prepared randomly from these bulk seed items. 

### 2.2. Production of Oil and Flour Samples

The seed oil was pressed from 600 g seed samples of each cultivar for both years in the laboratory of Slopmax Kft. (Budapest). Cold oil pressing was carried out using a Quatros laboratory hydraulic oil press with a self-designed stainless steel sample container. The maximum pressure was 10 metric tons. Each raw poppy seed material was pressed without any pre-treatment resulting in 300 mL of oil per sample. After the oil pressing, the residue (pellet) from all samples was milled with a Clatronic KSW 3306 R electric coffee mill operated with stainless steel beater blade to obtain a natural seed flour. Until the sensory test, the flour was stored in paper bags at room temperature while the oils were stored in glass bottles in the fridge (+4 °C). Oil pressing and flour production were conducted in January 2023 for all samples.

### 2.3. Sensory Test

Sensory tests were implemented in the Sensory Laboratory of MATE in February 2023. The panellists worked in individual booths designed in accordance with ISO 8589 [18]. Samples were labelled with 3-digit random number codes and were served in a randomized order. Panellists used still mineral water for palate cleansing. In case of poppy seed flour, the flour was served with cooked paste as a carrier. In case of oil samples, we used disposable plastic cups; for flour samples, we applied glass bowls. Samples were evaluated by sensory profile analysis according to ISO 13299 [19]. The sensory attribute list was generated based on a literature research and a pre-session before testing, where the most relevant attributes were recorded for both the oil and the flour samples. The attributes were the following for the oil samples: colour hue, odour intensity, poppy odour, overall flavour, poppy flavour, taste persistency and throat catching. In case of poppy seed flour, the list included colour hue, granularity, oily appearance, tactile perception, odour intensity, poppy odour, overall flavour intensity and poppy flavour. All attributes were measured on unstructured scales. To support the evaluation procedure, one of the samples was used as a reference. The reference values were established during the pre-test session. Due to the high number of total samples, only a maximum of six samples were tested in a single session. Panellists had experience in the quality evaluation of horticultural and agricultural products. A test session was implemented by a sensory-analysis-supporting software, ProfiSens, which is an own developed software of the Sensory Laboratory of MATE.

### 2.4. Determination of Fatty Acid Content and Composition of the Oil and Flour Samples

The fresh poppy seed oil and flour samples were analysed in MATE laboratory, Kaposvár. The oil content was determined by Soxhlet extraction according to the Hungarian standards MSZ ISO 7009:1983 [20] (measuring the oil content of oil seeds). The fatty acid composition of the oils was analysed by the capillary gas-chromatographic method and identification of the constituents was carried out with comparison of retention times with those of standard compounds, according to the standard MSZ EN ISO 12966-2 [21] (animal and vegetable fats: gas-chromatographic determination of fatty acid methyl esters).

### 2.5. Determination of the Volatile Compounds of the Oil

The volatile fraction of the oil has been determined by the headspace SPME method right after production. Sampling was performed by solid-phase microextraction applying a Supleco 24 Ga, 2 cm, 50/30 μm DVB/CAR/PDMS Stableflex fiber directly on the headspace of 2 mL of poppy seed oil at room temperature for 30 min. This fiber type has already been tested and advised by Krist and her co-workers [12] in their previous study. After sampling the manual SPME holder, it was taken immediately into the GC-MS instrument. Volatile compounds were separated using 30 m × 0.25 mm HP-5-MS nonpolar column (film thickness 0.25 µm) using a GC 6890 N, detector: 5975 Inert mass selective detector (Agilent Technologies). The injector temperature was 230 °C in splitless mode. Oven temperature was programmed as follows: the initial temperature was 60 °C, then it was raised by 3 °C/min until 240 °C and held for 5 min. The ionization voltage was 70 eV. The carrier gas was helium with a constant flow of 1 mL/min. 

Compounds were identified by comparing their mass spectra to those of authentic compounds as well as by using mass spectra libraries (NIST, Wiley). Linear retention indices (LRI) were determined based on *n*-alkane hydrocarbons analyzed before in the same GC-MS conditions using the method of Van Den Dool and Kratz [22]. LRI values were also compared to the authentic compounds as well as to the previous literature data.

### 2.6. Statistical Analysis

Sensory data were analysed by ProfiSens. One-way analysis of variance and pairwise significant differences were calculated. Differences among the varieties were also evaluated by Hierarchical Cluster Analysis (HCA) and the correlations among the investigated sensory characteristics and all biochemical parameters by Categorical Principal Analysis (CATPCA) using the software SPSS version 27. In case of the Pearson correlations, we evaluated the values as follows: strong (r ≥ 0.9); moderate (0.7 ≤ r < 0.9) and good (0.6 ≤ r < 0.7).

## 3. Results and Discussion

### 3.1. Sensory Test

**Figure 2:**
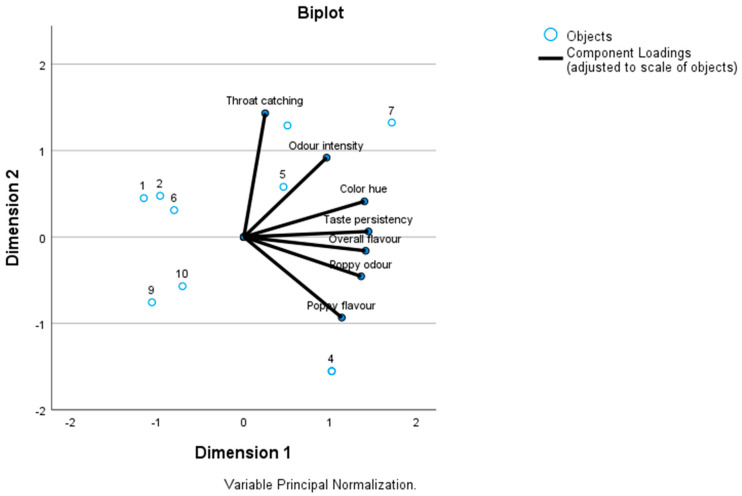
Sensory data biplot of poppy seed oil samples 1 ‘Albakomp’ 2021; 2 ‘Orel’ 2021; 3 ‘Zeno Plus’ 2021; 4 ‘Zeno Plus’ 2022; 5 ‘Korona’ 2021; 6 ‘Korona’ 2022; 7 ‘Morgana’ 2021; 8 ‘Morgana’ 2022; 9 ‘Snow White’ 2021; 10 ‘Snow White’ 2022.

Figure 2 shows the biplot of sensory data of CATPCA. The samples originated from two consecutive harvest years. In case of ‘Zeno Plus’ and ‘Snow White’, the two sets of oil samples from the two years are overlapping; ‘Korona’ and ‘Morgana’ samples show a larger difference, while the samples are located close to each other. Attribute vectors show that taste persistency and overall flavour values followed similar patterns. Poppy odour and poppy flavour also point to the same direction. ‘Zeno Plus’ samples showed the highest intensities in poppy odour and poppy flavour, while ‘Albakomp’, ‘Orel’ and ‘Snow White’ had moderate values here. Both samples of the high morphine containing the ‘Morgana’ variety had the highest intensity in throat catching attribute while ‘Zeno Plus’ was advantageous also from this respect. Differences in flavour profiles of spring and winter poppy varieties have been shown earlier [15], but the present study demonstrates it also between high-alkaloid (industrial) and low-alkaloid (culinary) cultivars. 

**Figure 3:**
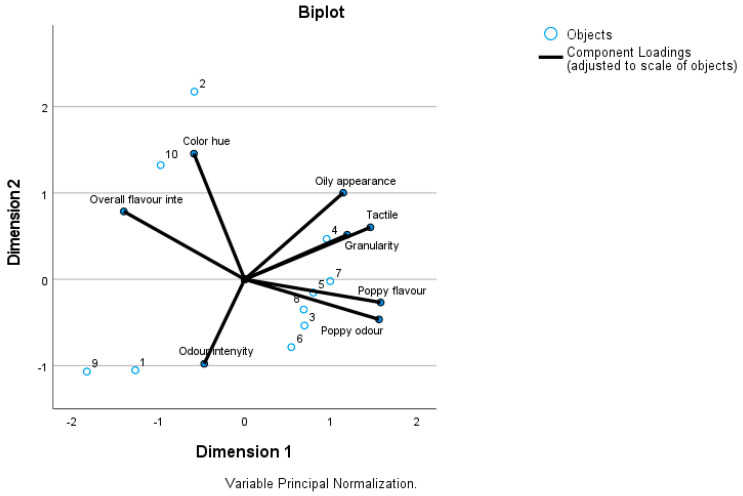
Sensory data biplot of poppy seed flour samples 1 ‘Albakomp’ 2021; 2 ‘Orel’ 2021; 3 ‘Zeno Plus’ 2021; 4 ‘Zeno Plus’ 2022; 5 ‘Korona’ 2021; 6 ‘Korona’ 2022; 7 ‘Morgana’ 2021; 8 ‘Morgana’ 2022; 9 ‘Snow White’ 2021; 10 ‘Snow White’ 2022.

Figure 3 shows the sensory data matrix CATPCA biplot of poppy seed flour samples. From the distribution of the samples, it is clearly visible that the varieties with white seeds (‘Albakomp’, ‘Orel’ and ‘Snow White’), are located on the left-hand side of the plane showing a low overall flavour intensity. The two harvest years of ‘Zeno Plus’ are relatively distant from each other, while in the case of ‘Korona’ and ‘Morgana’, the samples are quite close to each other. The distance between the two years of ‘Snow White’ is also considerable. Generally, poppy odour and poppy flavour are more typical to the blue-seed varieties. Nevertheless, the colour might have an indirect influence on the panel members which was unavoidable in this study. Attributes of tactile perception and granularity are overlapping each other, as these two attributes were showing similar data patterns.

**Figure 4:**
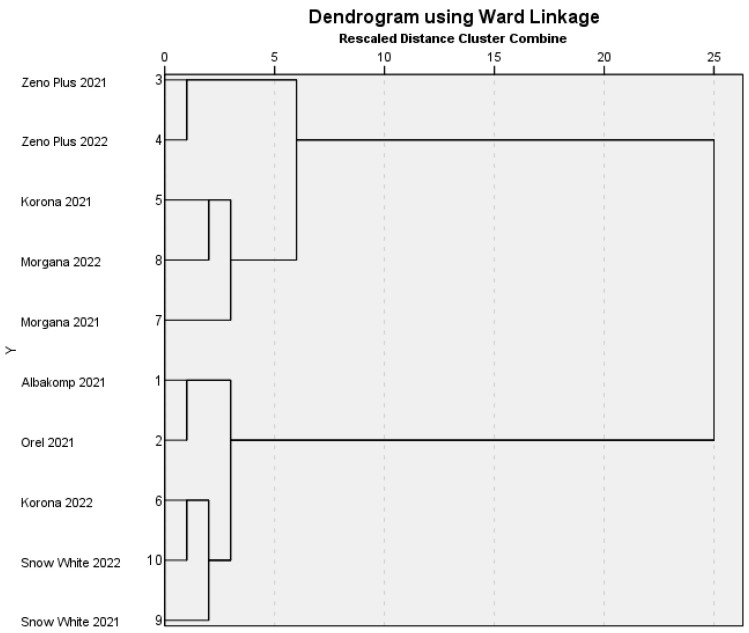
Hierarchical cluster analysis of poppy seed oil samples.

We also performed a Hierarchical Cluster Analysis to identify the similarities and differences between the sensory data matrices. Figure 4 shows the dendrogram of the oil samples.

From the cluster combination protocol, we can understand that for most varieties, the harvest year caused no relevant differences in the samples. ‘Zeno Plus’, ‘Morgana’ and ‘Snow White’ samples are assigned to the same clusters, independent from the harvest year. Only in the case of ‘Korona’, there was a larger difference between the two years. It suggests that this variety may be more sensitive to the weather conditions and/or to the storage time. The effect of the environmental conditions is well known to the yield and alkaloid content of poppy [4]; however, according to our knowledge, the effect of the year on sensory characteristics has not been demonstrated in this species until now.

**Figure 5:**
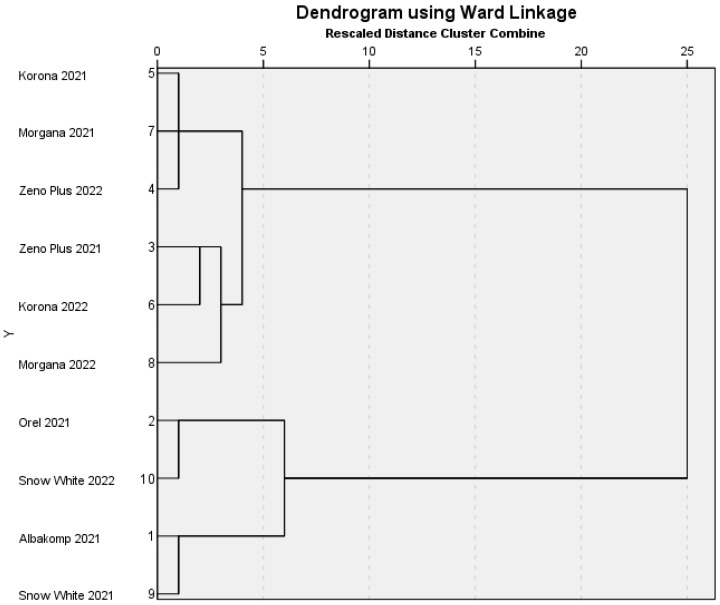
Hierarchical cluster analysis of poppy seed flour samples.

The cluster analysis of the flours (Figure 5) shows two main clusters, where the major clustering factor is the colour of the seed. White-seeded varieties (‘Albakomp’, ‘Orel’ and ‘Snow White’ samples) are assigned to the first cluster, while the blue-seeded samples belong to the second. If we formulate three clusters, then ‘Albakomp’ 2021 and ‘Snow White’ 2021 are in the first cluster, ‘Orel’ 2021 and ‘Snow White’ 2022 are in the second, and the blue varieties are in the third one. When considering the fourth cluster, there is a smaller division among the blue varieties. Interestingly, flour samples from different years of the same variety are not so close to each other than in the case of the oils.

### 3.2. Fatty Acid Composition

A total of eleven fatty acids were detected in the poppy oils, and the results are listed in Table 1. Ratios of polyunsaturated fatty acids (PUFA) in the tested poppy variety oils varies between 64.55–76.48% of the oil. The PUFA of culinary varieties (‘Snow White’, ‘Orel’, ‘Albakomp’ and ‘Zeno Plus’) is more than 6% higher (74.53% as mean) than that of the industrial varieties ‘Morgana’ and ‘Korona’ (68.28% as a mean). The essential ω-6 linoleic acid (C18:2) accumulated in 71.5% and the ω-3 linolenic acid (C18:3n3) in 0.50% as the variety mean. Among the monounsaturated fatty acids (C16:1, C18:1n9, and C20:1), oleic acid is present in the highest percentages (12.68–23.37). 

The monounsaturated fatty acid (MUFA) content of the industrial varieties ‘Korona’ and ‘Morgana’ varies between 19.11% and 23.63%, while in the culinary varieties, it is only 12.87–15.92%. Among the saturated fatty acids, palmitic (C16:0) and stearic acids (C18:0) are present in the highest percentages in the oils with a balanced ratio between the food and industrial varieties. Principally, differences among the industrial and culinary varieties in their fatty acid composition might be based on common origin during breeding or a genetic linkage might exist between fatty acids and alkaloid content. Interestingly, neither the former fact seems to be likely [23], nor are there any proofs known for the latter statement.

The composition of the main fatty acids also showed differences between white- and blue-seed varieties. As the main feature, we could establish that oleic acid (18:1) showed higher percentages in the blue varieties while linoleic acid (18:2) was accumulated in higher ratios in the white seeds. At the same time, the saturated fatty acid (SFA) content of both types of seeds did not differ significantly. Rokosik et al. [24] also determined a similar pattern between the poppy seeds of different colours, although no variety names have been mentioned in that paper. It would be worth detecting in the future if the genetic relationship might explain the specific fatty oil spectrum of the two seed colour groups.

As for the density of the oil, no difference among the varieties has been established.

The oil content of the poppy flour is between 27.5–37.2%. The lowest concentration was detected in the seeds of ‘Albakomp’ and ‘Korona’ 2022 (both below 30%), while the highest amount was measured in ‘Orel’ and ‘Korona’ 2021 which reflect a higher effect of the year than that of the genotype (industrial or food variety, blue- or white-seeded cultivar) in this case. The fatty acid composition of poppy flours (Table 2) is rather similar to the results of the poppy oil (Table 1). 

Recently, several research groups have analyzed poppy seed samples of various origins for oil content and fatty acid composition [11,25,26,27,28,29,30,31,32,33]. According to them, the oil content of the seeds is varying between 27.71 and 52.40%, depending on the variety, location, and extraction technology. Our data fit into the lower level of this range.

Based on the above references, the dominant fatty acid in poppy oil is linoleic acid, the ratio of which varies between 53% and 76% of the oil. Among the saturated fatty acids, palmitic acid (hexadecanoic acid) and stearic acid (octadecanoic acid) are present in 8–19% and 2–4%, respectively. In addition to these, it also contains oleic acid (13–25%) and a lower amount of linolenic acid (0.24–1.32%). Thus, the detected fatty acid composition of the experimental varieties is in accordance with the literature, defining linoleic, oleic, and palmitic acids as the most abundant fatty acids in poppy oil [15,25,26,27,28,29,30,31,32,33]. The measured percentages of linoleic acid fits into the upper range of the international data. The finding of Nergiz and Ötles [25], Azcan et al. [26], and Lančaričová at al. [30] that the white-seeded variety is characterized by increased levels of palmitic acid and linoleic acid, as well as a lower content of stearic, oleic, and linolenic acid compared to the blue poppy, could not be proved in our study. As these authors investigated Turkish and Slovakian accessions while ours were of Hungarian origin (except the Czech ‘Orel’), it seems to be more likely that the oil spectrum is associated with genotype independent from the seed colour.

### 3.3. Volatile Composition 

In the analysed poppy varieties’ oils, 28 different headspace volatile components were detected, and the main ones are shown in Table 3.

Hexanol, n-hexanal, pentanol and acetic acid were detected in all samples which is in accordance with the previous literature references [12,13,14,34]. However, Luhmer et al. [15] detected caproic acid as the dominant volatile in 12 out of 13 samples, which is not a main component in our samples. The highest proportion of it was detected in ’Zeno Plus’ in year 2021 (13.88%). Interestingly, in case of ’Morgana’, this compound was absent in 2021 but present at a proportion 5.17% in 2022. Thus, the significant difference in the measured values of volatile compounds can be observed not only among the varieties, but also between the years of cultivation. A further example for this phenomenon is acetic acid, which reached 68.17% in ‘Korona’ 2022 and 25.11% in ‘Snow White’ 2022 samples, while it was only 8.91% and 4.64%, respectively, in the former year.

Pentanol was also present in larger quantities in the plants grown in 2022, except for the noscapine-containing ‘Korona’ variety. On the other hand, a typical genotype-dependent characteristic seems to be the presence of 2 and 3 methyl-butanal and 3 methyl-butanol only detected in both years of the ‘Morgana’ industrial variety. Finally, the proportions of pentane and 2-pentylfuran showed large fluctuations among varieties.

### 3.4. Correlation among Sensory and Phytochemical Values

The correlations among the analysed attributions in case of the oils and the flour samples are demonstrated in Tables S1 and S2, respectively. In these results, we highlight, especially, the connection between the experienced sensory features and the analytical composition of the samples.

The sensory features of the oil samples are influenced both by fatty acid and volatile compositions, to some extent. There was a strong negative connection detected between saturated fatty acid ratio (SFA) and all the flavour and odour characteristics. Correlation of the individual SFAs with these features is also strongly negative, except stearic acid, where the correlation was positive. The throat-catching feeling seems to be in connection with the total polyunsaturated fatty acid (PUFA) content and with that of linoleic acid. As the latter one is the main component of each poppy oil also having beneficial effects for human health, the throat-catching property may only be decreased but not eliminated. The throat-catching effect was in moderate connection also with other chemical constituents, either positively (e.g., arachidonic acid) or negatively (e.g., elaidinic and oleic acids). 

From the headspace volatile compounds, 2-pentylfuran demonstrated a strong negative effect on each of the sensory characteristics except throat-catching feeling. As it was found that its presence varies among genotypes (Section 3.3), this feature presumably might be improved by breeding. Similarly, a negative but moderate connection was detected between the sensory odour and flavour traits, and the concentration of hexanal. In parallel, the presence of 2-pentanone and hexanol contribute positively to these features (good and moderate correlations). In general, straight-chain alcohols and aldehydes like hexanal and hexanol are known as “green leafy odour” volatiles, which do not seem very advantageous for poppy aroma [35]. Besides, among the volatiles, γ-n-caprolactone and pentane seem to be in connection with the throat-catching property of the oil.

Somewhat surprisingly, oil density was shown negatively correlating with colour, odour and flavour values. Nevertheless, differences in density of the samples are not significant. 

The oil content of poppy flour has a positive effect on several sensory properties, like colour, appearance, tactility and poppy flavour. On the other hand, the ratio of total ω-3 fatty acid and especially that of α-linoleic acid show strong negative correlations with poppy odour and moderately negative ones with poppy flavour. However, both contribute positively to the overall flavour intensity. Odour intensity is in good positive correlation with both margaric acid and eicosenoic acid ratios, although they are present in the poppy oils only in low concentrations.

## 4. Conclusions

We could establish that characteristic differences are manifested among the poppy genotypes concerning the evaluated sensory features of their oil and flour samples. It seems, however, that intensities in poppy odour and poppy flavour are mainly not influenced by the type of the variety (industrial or culinary one) but more with the seed colour. Blue-seed varieties demonstrated a bigger preference in the sensory tests than the white-seed ones. However, further studies are necessary to clarify whether it is a direct or an indirect connection. Only in the throat-catching attribute could we state that industrial varieties showed higher intensity. Referring to the effect of the cultivation years, it could be seen that this parameter had a small effect on the sensory characteristics of the varieties.

The fatty oil composition profile of the tested varieties is rather similar, with the exception of the polyunsaturated fatty acids which have been detected above 70% only in the culinary varieties. The same pattern can be seen in the case of the main fatty acid compound, linoleic acid. For the human diet, it is an advantageous situation, although—according to our knowledge—these varieties have not been selected especially for this trait. 

The spectrum of the headspace volatiles showed much larger differences both among the tested varieties and between samples originating from the two vegetation years. A higher ratio of saturated fatty acids negatively influenced the flavour and odour characteristics which is in coincidence with the health-beneficial effects of the unsaturated ones. In case of using the poppy cake flour, a higher rest oil content may contribute to better colour, appearance, tactility and flavour, which should be taken into consideration during pressing.

The presented data contribute to a goal-oriented breeding and use of poppy varieties in order to increase both the compositional quality and the customers’ acceptance of healthy food and culinary products of this species.

## Figures and Tables

**Table 1:** The fatty acid composition (% of total oil) and density of poppy oil of the samples originating from different poppy varieties and vegetation years.

Variety/Content	Poppy Seed Oil Density g/cm^3^	C16:0 Palmitic Acid	C16:1 Palmitoleic Acid	C17:0 Margaric Acid	C18:0 Stearic Acid	C18:1n9 Oleic Acid	C18:2 Linoleic Acid	C18:3n3 Linolenic Acid	C20:0 Arachinic Acid	C20:1 Eicosenoic Acid	C20:2 Eicosadienoic Acid	C22:0 Behenic Acid
Snow White 2021	0.91	8.50	0.12	0.05	2.10	13.15	75.40	0.45	0.11	0.08	0.03	0.02
Snow White 2022	0.91	8.63	0.13	0.05	2.30	14.83	73.40	0.45	0.10	0.07	0.03	0.01
Albakomp 2021	0.92	9.89	0.15	0.05	2.13	15.68	71.28	0.55	0.13	0.09	0.03	0.02
Orel 2021	0.90	8.60	0.11	0.04	2.32	13.09	75.09	0.50	0.12	0.07	0.03	0.02
Zeno Plus 2021	0.91	8.33	0.13	0.05	2.38	15.47	72.93	0.45	0.12	0.08	0.03	0.02
Zeno Plus 2022	0.91	8.22	0.12	0.05	2.24	12.68	75.91	0.53	0.12	0.07	0.03	0.02
Korona 2021	0.92	8.00	0.11	0.04	2.55	19.14	69.32	0.54	0.15	0.10	0.02	0.02
Korona 2022	0.91	7.79	0.10	0.04	2.22	18.90	70.15	0.52	0.12	0.10	0.03	0.02
Morgana 2021	0.90	9.23	0.16	0.05	2.28	20.07	67.46	0.49	0.14	0.08	0.02	0.02
Morgana 2022	0.91	9.20	0.17	0.04	2.39	23.37	64.02	0.51	0.16	0.09	0.02	0.02
Average%	0.91	8.64	0.13	0.05	2.29	16.64	71.50	0.50	0.13	0.08	0.03	0.02

**Table 2:** The oil (% of d.w.) and fatty acid composition (% of total oil) of poppy flour of the samples originating from different poppy varieties and vegetation years.

Variety/Content	Oil Content %	C16:0 Palmitic Acid	C16:1 Palmitoleic Acid	C17:0 Margaric Acid	C18:0 Stearic Acid	C18:1n9 Oleic Acid	C18:2 Linoleic Acid	C18:3n3 Linolenic Acid	C20:0 Arachinic Acid	C20:1 Eicosenoic Acid	C20:2 Eicosadienoic Acid	C22:0 Behenic Acid
Snow White 2021	31.10	8.33	0.12	0.05	2.37	13.84	74.34	0.58	0.18	0.10	0.04	0.05
Snow White 2022	32.20	8.63	0.14	0.06	2.60	13.18	74.28	0.72	0.20	0.09	0.03	0.06
Albakomp 2021	27.50	9.61	0.15	0.05	2.42	16.59	70.03	0.71	0.21	0.12	0.03	0.07
Orel 2021	37.20	8.34	0.11	0.04	2.48	13.57	74.38	0.72	0.18	0.09	0.04	0.05
Zeno Plus 2021	31.20	8.33	0.12	0.05	2.37	13.84	74.34	0.58	0.18	0.10	0.04	0.05
Zeno Plus 2022	33.00	8.44	0.12	0.05	2.56	16.04	71.97	0.51	0.15	0.09	0.03	0.04
Korona 2021	35.00	8.26	0.11	0.05	2.70	20.04	67.85	0.61	0.19	0.12	0.03	0.05
Korona 2022	28.90	8.11	0.10	0.05	2.48	20.30	67.92	0.63	0.19	0.13	0.04	0.05
Morgana 2021	32.30	9.45	0.16	0.05	2.37	20.60	66.43	0.60	0.18	0.09	0.03	0.04
Morgana 2022	34.90	9.66	0.17	0.05	2.56	24.11	62.44	0.61	0.21	0.10	0.03	0.05
Average %	32.33	8.87	0.13	0.05	2.49	17.21	70.40	0.63	0.19	0.10	0.03	0.05

**Table 3:** The main volatile compounds (% of GC area) of the seed oils.

Component	RT	LRI	Albakomp 2021	Korona 2021	Korona 2022	Morgana 2021	Morgana 2022	Orel 2021	Snow White 2021	Snow White 2022	Zeno Plus 2021	Zeno Plus 2022
pentane	1.5	520	0	3.15	0	0	0	11.29	2.12	3.11	1.12	0.29
acetic acid	1.77	646	6.51	8.91	68.17	6.21	7.12	3.91	4.64	25.11	9.66	0.95
3-methyl-butanal	2	652	1.62	0	0	19.97	1.17	0.11	0	0	0.12	0
2-methyl-butanal	2.04	661	0	0	0	7.66	0.86	0	0	0	0.11	0
3-methyl-butanol	2.48	685	0	0	0	29.75	1.05	0.33	0	0	2.61	0
2-pentanone	2.54	688	5.01	0	0	0	0	0	0.36	0	0	49.06
pentanol	2.78	763	3.84	7.09	3.06	5.72	8.44	6.97	1.37	13.03	2.87	3.67
n-hexanal	3.1	797	51.75	54.55	15.34	17.01	57.7	34.95	67.37	52.41	52.38	15.51
hexanol	4.21	865	18.21	6.39	0.73	1.17	8.66	6.25	13.86	1.26	5.54	12.35
heptan-2-one	4.6	888	0	0	0	0	2.5	7.61	0	0.83	1.09	8.52
2-pentylfuran	7.07	997	0	2.48	0	0.79	3.78	13	0.56	0	3.4	2.11
caproic acid	7.29	1002	0	4.48	2.2	0	5.17	1.8	0	0	13.88	4.66
gamma-n-caprolactone	9.55	1066	0	0.88	0	0	0	1.29	0	0	0	0

## Data Availability

The data presented in this study are available on request from the corresponding author.

## Supplementary Materials

The following supporting information can be downloaded at: https://www.mdpi.com/article/10.3390/foods12173165/s1, Table S1: Correlations among the analysed attributions in case of the oils and Table S2: Correlations among the analysed attributions in case of the flour samples.
